# An Emerging Role for Long Non-Coding RNA Dysregulation in Neurological Disorders

**DOI:** 10.3390/ijms141020427

**Published:** 2013-10-14

**Authors:** Chiara Fenoglio, Elisa Ridolfi, Daniela Galimberti, Elio Scarpini

**Affiliations:** Department of Pathophysiology and Transplantation, University of Milan, “Dino Ferrari” Center, IRCCS Cà Granda Foundation Ospedale Maggiore Policlinico, Via F.Sforza 35, Milan 20122, Italy; E-Mails: elisa.ridolfi@unimi.it (E.R.); daniela.galimberti@unimi.it (D.G.); elio.scarpini@unimi.it (E.S.)

**Keywords:** lncRNA, RNA, neurodegenerative diseases, epigenetics

## Abstract

A novel class of transcripts, long non coding RNAs (lncRNAs), has recently emerged as key players in several biological processes, including dosage compensation, genomic imprinting, chromatin regulation, embryonic development and segmentation, stem cell pluripotency, cell fate determination and potentially many other biological processes, which still are to be elucidated. LncRNAs are pervasively transcribed in the genome and several lines of evidence correlate dysregulation of different lncRNAs to human diseases including neurological disorders. Although their mechanisms of action are yet to be fully elucidated, evidence suggests lncRNA contributions to the pathogenesis of a number of diseases. In this review, the current state of knowledge linking lncRNAs to different neurological disorders is discussed and potential future directions are considered.

## 1. Introduction

**Figure 1:**
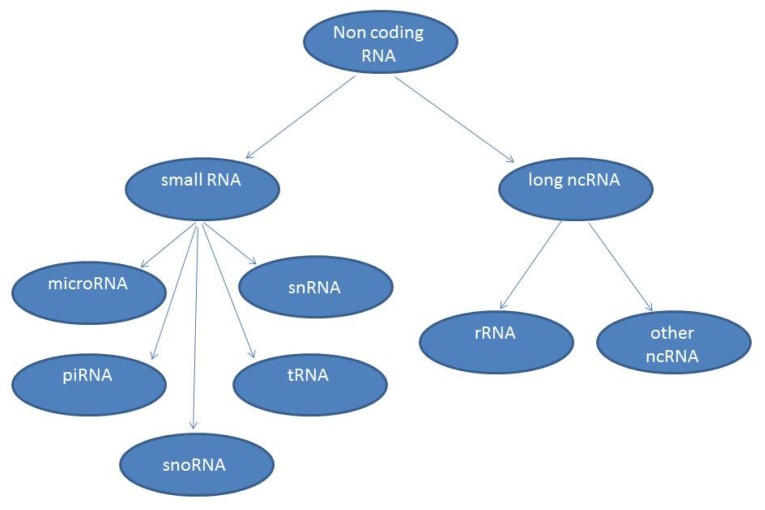
Non-coding RNAs (ncRNAs) are arbitrarily grouped basing on size. Small ncRNAs being less than 200 nucleotides and long ncRNAs greater than 200 nucleotides.

For the last few decades of the 20th century, the underlying dogma of molecular biology has been that the purpose of RNA is to direct the assembly of proteins from amino acids. A few exceptions to this paradigm were known (such as ribosomal RNA and transfer RNA, which are functional RNA macromolecules that do not code for protein). Non-coding RNAs (ncRNAs) include the familial “housekeeping” RNAs and the thousands of regulatory RNAs that are the subject of recent intense investigation. NcRNAs come in many different sizes and for this reason have been divided into small and long classes: small ncRNAs (sncRNA) being less than 200 nucleotides (nt), and long ncRNAs (lncRNA) greater than 200 nt to over 100 kb in length [1]. The current cut-off is arbitrary and corresponds to certain biochemical fractionation protocols and excludes most categories of small infrastructural or regulatory RNAs (tRNAs, snRNAs, miRNAs, siRNAs, piRNAs, tiRNAs, spliRNAs, sdRNAs and others, Figure 1).

An estimation of the number of lncRNA loci from transcriptional surveys in mammals suggests that they are at least as numerous as protein-coding genes [2] with many of lncRNAs identified in intergenic regions alone [3]. With a few exceptions, it is only within the past few years that the functions and mechanisms of lncRNAs have begun to emerge.

The lncRNAs that have been studied in detail were found to be involved in different biological processes including X chromosome inactivation, nuclear structure, genomic imprinting and development.

In the following paragraphs some of the known functions of lncRNAs will be discussed with particular regard to their role in neurological disorders.

## 2. Identification of lncRNAs

Data derived from massive cloning and traditional sequencing methods have demonstrated that mammalian genomes produce thousands of RNA transcripts in addition to protein-coding genes [2]. These studies modified our simplistic view of the genome and suggested the presence of thousands of previously unknown transcripts. Recently more than 3000 intergenic ncRNAs have been found in the mammalian genome, by using information from chromatin modifications [3,4]. Evidence gained from ChIP-Seq analyses demonstrated that transcribed protein-coding genes have unusual chromatin modifications; these modifications are trimethylation of histone H3 at lysine 4 (H3K4me3) at the promoter region and trimethylation of histone H3 at lysine 36 (H3K36me3) in the body of the gene [5]. By eliminating these chromatin domains that correspond to protein-coding genes more than 3000 intergenic domains transcribed into lncRNAs have been found. Bioinformatics analyses showed that the majority of these transcripts have no protein-coding capability. Moreover, it was shown that many of these lncRNAs are able to interact with multiple chromatin-modifying complexes in different human and mouse cell types suggesting that lncRNAs may be involved in epigenetic regulation [3].

Recent advances in RNA sequencing technology (RNA-Seq) addressed further interrogation of the total cellular RNA, or, transcriptome, at a much higher resolution [6]. Thousands of transcripts, in addition to protein-coding mRNAs and microRNAs, have been found to be expressed in a wide range of tissues and cell types [7].

## 3. Epigenetics

Many of the lncRNAs seem to be involved in epigenetic mechanisms of gene regulation. Although the field of epigenetics earned its name over 50 years ago, just in this past decade the significance of epigenetics has been recognized in human health and disease [8]. The term epigenetics refers to changes in gene expression and/or phenotype that can be heritable without a change in the underlying DNA sequence. Several factors contribute to epigenetic mechanisms of gene regulation including DNA methylation, histone modifications, and ncRNAs. DNA methylation in the promoter region of genes is typically associated with transcriptional repression. Several mammalian enzymes are responsible for establishing and maintaining DNA methylation in the genome [9].

The modification of histone proteins has also been found in epigenetic regulation [10]. Histones are highly conserved proteins that package DNA in the nucleus and modulate the accessibility of transcription factors and RNA polymerases to DNA. Histone modifications typically take place at amino acids located in the *N*-termini of histones such as lysine, arginine and serine residues that can be acetylated or methylated (lysine and arginine) and phosphorylated (serine). Histone modifications are placed on and removed from histone residues by numerous enzymes that usually work as part of multi-protein complexes [10].

A relevant question in biology is how chromatin-modifying complexes are targeted to specific genomic loci since many of these enzymes lack DNA binding capacity. Recent studies suggest a potential role for ncRNA in driving chromatin-modifying complexes to genomic loci [11–13], however, the exact mechanism by which ncRNAs drive complexes is not known and is currently under investigation.

## 4. Functions of lncRNAs

Basing on our current knowledge of lncRNAs, it appears that such molecules are involved in many different aspects of cellular functions (reviewed in [14,15]). The roles of lncRNAs in the regulation of gene expression and organismal development are different and just beginning to be discovered. Biological processes dependent upon lncRNAs include imprinting and gene dosage regulation, stem cell pluripotency, embryonic development and segmentation, hematopoiesis, and neural cell fate determination (see for review ref [16]). LncRNAs may employ a number of mechanisms to impact gene expression via *cis* and *trans* processes.

### 4.1. Gene Imprinting

While the function of parental gene imprinting is still unclear, lncRNAs have been found to participate in imprinting processes. These are referred to as the events that influence the monoallelic expression of a gene according to its parents of origin. Imprinting control regions (ICRs) are DNA regions that are differentially methylated depending on their parental origins. Unmethylated ICRs cause specific expression of nearby lncRNAs, which then suppress neighboring genes in *cis*. Several imprinted clusters contain protein-coding genes and lncRNAs that are reciprocally expressed, such as *IGf2r*/*Air* [17], *Dlk1*/*Gtl2* [18,19], and *Nesp*/*Nespas*/*Gnas* [20]. Some of these lncRNAs can exert their function by recruiting epigenetic factors, such as PRC2 and G9a, in order to control the imprinted expression of neighboring coding genes [21,22].

Airn and Kcnq1ot1 are examples of lncRNAs that cause suppression of paternally inherited genes. Kcnq1ot1, in particular, is involved in the repression of several protein-coding genes in *cis* through interaction with repressive chromatin modifying complexes [23,24].

H19 was one of the first mammalian lncRNAs to be identified and is highly expressed in the embryo [25,26]. Though it does not seem to act as an lncRNA [27], H19 likely functions as an miRNA precursor [28,29] and is mutually imprinted with the protein-coding gene *Igf2*.

### 4.2. Gene Dosage and X Chromosome Inactivation

The X chromosome inactivation (XCI) indicates the mechanisms by which the difference in X-linked gene dosage between XX females and XY males is exerted in therian mammals in which one of the two X chromosomes in females is silenced (the inactive X or Xi) so that only one X remains active and is expressed in each female cell (the active X, or Xa) [30]. It is well-known that XCI in placental mammals is largely controlled by a cluster of lncRNA loci known as the X-inactivation center (*Xic*) [31]. The X (inactive)-specific transcript (*Xist*) is highly expressed from Xi during the onset of *XCI* but not from *Xa*, contributing a defining moment for the realization that lncRNAs can have profound roles in the control of gene expression even though the exact mechanism of action is still not completely understood. Some evidence suggests that Xist mediates the chromosome X silencing effects by interacting with repressive chromatin-modifying complexes such as PRC2 [22].

*Xist* itself is also regulated by other lncRNAs. Initially, *Xist* and *Tsix*, its antisense transcript, transcribed from a promoter downstream of *Xist*, are expressed on both X chromosomes. However, *Tsix* expression continues on the X that will remain active (*Xa*) and this activity recruits dnmt3a to suppress *Xist* from being transcribed on *Xa* [32]. Conversely on *Xi*, it is Tsix that is suppressed, potentially through another lncRNA that is part of the X inactivation center, Jpx [33]. With *Tsix* suppressed, the protein PRC2 is recruited to induce histone modification marks at the 5′ end of *Xist*. This upregulates *Xist* expression on *Xi* and causes further propagation of these silencing marks throughout Xi, which are maintained across the lifetime of the organism [33].

### 4.3. Embryonic Development and Segmentation

LncRNAs are likely implicated in processes involving animal development. The *Hox* genes encode homeodomain TFs that are crucial for anterior-posterior pattern formation in bilateral metazoans [34]. *Hox* genes are structured in linear clusters along the chromosome, and mammals have four paralogous clusters, *HoxA*, -*B*, -C, and -D. Several lncRNAs are encoded within these clusters, including HOTAIR (Hox antisense intergenic RNA) from *HoxC*, and HOTTIP and Mistral from *HoxA* [35–37]. The expression of *Hox* genes is also regulated by lncRNAs [36]. Some Hox-related lncRNAs operate in *cis*, having either enhancing or repressive effects. However, some like the human HOTAIR works in *trans*, and it is expressed from the *Hox* locus marking a boundary of active and inactive chromatin [35]. Furthermore, HOTAIR, similar to *Xist*, interacts with chromatin-modifying complexes such as PRC2 and the corepressor complex CoREST [35] and may guide these complexes to genomic loci. Overexpression of HOTAIR caused cells to become metastatic when injected into mice compared to control cells with an empty vector. Moreover, HOTAIR may serve as a scaffold for targeting chromatin-modifying complexes to chromatin [35].

Recently HOTAIR was found to be crucial for cell growth and viability and that its knockdown induced apoptosis in breast cancer cells Moreover it was found that HOTAIR is transcriptionally induced by estradiol [38]. It is possible that other lncRNAs that interact with chromatin-modifying complexes also function in a manner similar to HOTAIR.

### 4.4. Stem Cell Pluripotency and Cell Fate Determination

The promoters of more than 100 lncRNAs are bound by stem cell factors. Disruption of these lncRNAs can alter cell differentiation. The human lncRNA-RoR (RoR) is a recently identified lncRNA that is capable of reprogramming differentiated cells to induce pluripotent stem cells [39,40]. RoR is highly expressed both in embryonic stem cells and in induced pluripotent stem cells, due to the regulation of RoR by pluripotency transcription factors such as Oct4, Sox2 and Nanog. Interestingly it was observed that knockdown of RoR leads to a modest increase in apoptosis and activation of p53 pathways [40]. Although the underlying mechanisms still remain to be fully clarified, Zhang *et al.* recently [41] demonstrated that human RoR is a strong negative regulator of p53 influencing the inhibition of p53-mediated cell cycle arrest and apoptosis.

Recently, a refined analysis from Guttman *et al.* [39] performed loss-of-function studies on 226 lncRNAs expressed in mouse embryonic stem cells characterizing the effects on gene expression. The authors identified 26 lncRNAs able to maintain the pluripotent status. In particular, knockdown of these lncRNAs resulted in a loss of pluripotency markers, and reduction of Nanog promoter activity. Simultaneously, expression patterns similar to differentiation into specific lineages were produced, suggesting that lncRNAs repressed differentiation programs in mouse embryonic stem cells. Altogether these findings support the hypothesis that some lncRNAs are integral members of a regulatory network, together with key pluripotent transcription factors, which modulate pluripotency and lineage-specific differentiation pathways in mouse embryonic stem cells.

lncRNAs are implicated in cell fate determination events in multiple cells lineages, including the nervous system. Taurine upregulated gene 1 (*TUG1*) is an lncRNA expressed in the developing retina and brain, as well as in adult tissues. It has been found that in the newborn retina, loss of TUG1 resulted in malformed or non-existent outer segments of transfected photoreceptors, thus suggesting that TUG1 is required for the proper formation of photoreceptors in the developing rodent retina [42]. Evf2 is instead a mouse lncRNA that appears to recruit Dlx and Mecp2 transcription factors to important DNA regulatory elements in the *Dlx5-Dlx6* intergenic regions and controlled Dlx5, *Dlx6* and Gad1 expression through *cis* and *trans* acting mechanisms. Evf2 mouse mutants appeared to have reduced numbers of GABAergic interneurons in early postnatal hippocampus and the dentate gyrus. This situation is restored to normality in Evf2 mutant adult hippocampus although reduced synaptic inhibition still occurred [43,44].

Although many lncRNAs have been shown to regulate gene expression only a few have been shown to have other cellular functions. For example, NEAT1 has been shown to play an important role in paraspeckle formation [45]. Also NRON has a role in nuclear import/export [46]. All together, these studies suggest that the lncRNAs have different cellular functions, many of which are yet to be identified and characterized for the mechanism of their function.

## 5. LncRNAs in Human Diseases

As the functions and mechanisms of lncRNAs are beginning to emerge, there is an intense interest in identifying any potential role of these molecules in human diseases. Several studies have shown that lncRNAs are dysregulated in human pathologies, however it has yet to be shown that these molecules are enough to drive the disease status.

lncRNAs have been strongly associated with cancer [47]. Recently, the lncRNA PCAT-1, was found to promote cell proliferation and is a target of PRC2 regulation, [48]. Moreover, ANRIL, which is upregulated in prostate cancer, is required for the expression of the tumor suppressors INK4a/p16 and INK4b/p15 [49]. HOTAIR upregulation is associated with poor prognosis in breast cancer [11], liver [50], colorectal [51], gastrointestinal [52] and pancreatic [53] cancers and probably contributes to increase also tumor invasiveness and metastasis [11].

MALAT-1, which is another lncRNA associated with various cancers and metastasis [54] was found to affect the transcriptional and post-transcriptional regulation of cytoskeletal and extracellular matrix genes [55]. Although lncRNAs have been extensively investigated in cancers several lines of evidence suggest a possible role also in different disease conditions such as cardiovascular diseases. For example, two lncRNAs have been found to be dysregulated in heart disease; the expression of the lncRNA MIAT is associated with increased risk of myocardial infarction, whereas the lncRNA ANRIL is associated with increased risk to coronary heart disease [56,57]. Recently, a novel lncRNA has been discovered, named DBE-T, that functions in *cis* and localizes to the Facioscapulohumeral muscular dystrophy (FSHD) locus. FSHD is the third most common myopathy and is predominantly caused by a contraction of specific repeats mapping on chromosome 4q35 [58]. It is suggested that DBE-T likely acts as a locus control element by promoting active chromatin domains and thus FSHD would be caused from lncRNA “promoter mutations” able to perturb DBE-T regulation [58]. Furthermore, a novel lncRNA seems to be involved in the pathogenic mechanisms underneath the HELLP syndrome (hemolysis, elevated liver enzymes, low platelets) that is a recessively inherited life-threatening pregnancy complication [59].

## 6. Role of lncRNAs in the Central Nervous System

Recent evidence demonstrate that lncRNAs contribute to the complex biological system organization and gene regulatory networks of the central nervous system (CNS), affecting brain patterning, neural stem cell maintenance, neurogenesis and gliogenesis, stress responses, and synaptic and neural plasticity.

Mercer and colleagues identified 849 lncRNAs (among the 1328 examined), that are expressed in the adult mouse brain and found that the majority were associated with specific neuroanatomical regions, cell types, or subcellular compartments [60]. A complementary study showed that over 200 of these lncRNAs are expressed in developing and adult mouse brain and are largely derived from genomic loci located proximal to protein-coding genes with similar expression profiles in the brain [61].

Guttman *et al.* discovered more than 1000 evolutionarily conserved intergenic lncRNAs in mouse by analyzing chromatin signatures from four mouse cell types [5]. A functional analysis of the expression of these lncRNAs revealed the presence of a “brain cluster” of lncRNAs that is associated with biological processes including hippocampal development, oligodendrocyte (OL) myelination, brain aging, CREB and PGC1-alpha transcriptional regulation, and GABAergic neuronal (GABAN), G protein coupled receptor and calcineurin signaling pathways. An additional study demonstrated that 169 lncRNAs are differentially expressed during the sequential processes of mouse ventral forebrain-derived neural precursor cells mediated lineage restriction, GABAN and OL lineage specification, progressive OL lineage maturation, and terminal differentiation including myelination [62].

Detailed analyses of specific lncRNAs, dynamically expressed in the CNS, reveal potential roles in mediating neural cell fate decisions. The Sox2OT lncRNA, which contains the *Sox2* gene within one of its introns and is subsequently transcribed in the same direction [63], is expressed in regions of constitutive adult neurogenesis [60]. Moreover, Sox2OT is dynamically regulated in CNS structures during development, where it may be responsible for modulating Sox2 expression [64]. The lncRNA Nkx2.2AS regulates Nkx2.2, a transcription factor that is critical for OL lineage specification. A recent study reported that forced expression of Nkx2.2AS in NSCs *in vitro* enhances their differentiation along the OL lineage, in part, by inducing an increase in Nkx2.2 mRNA levels [65].

LncRNAs also modulate synaptic plasticity and promote long-term changes in synaptic strength. The rodent-specific *BC1* and primate-specific BC200 lncRNAs, are selectively targeted to postsynaptic dendritic compartments, where they regulate local protein synthesis by repressing the initiation of translation through an eIF4A-dependent mechanism [66–69]. Similarly, NTAB is a lncRNA that is expressed in developing and adult rat brain, where it is also found in neuronal processes [70]. Another lncRNA, MALAT-1, is enriched in hippocampal neurons, where it regulates several serine/arginine splicing factors important for synapse formation, density and maturation [71].

### 6.1. Dysregulation of lncRNAs in Neurological Disorders

#### 6.1.1. LncRNAs Play a Role in the Pathophysiology of Several Neurological Disorders

Angelman syndrome (AS) is a neurodevelopmental disorder associated with genomic imprinting and characterized by severe neurologic abnormalities [72]. Ube3a*-*as is a lncRNA transcribed antisense to the maternally expressed *Ube3a* gene, mutated or deleted in AS, suggesting that Ube3a*-*as may repress paternal Ube3a expression. Other studies have shown that repression of Ube3a is dependent on Ube3a-as [73,74]. However, other data has demonstrated that silencing of paternal *Ube3a* can occur in the absence of Ube3a-as and implies a more complex regulatory relationship underlying the imprinting of *Ube3a* [75].

LncRNAs may influence the pathogenesis of fragile X syndrome (FXS), which is characterized by a triplet nucleotide repeat expansion in the 5′UTR of *FMR1*, the gene encoding the neuronal development protein, FMRP. The lncRNAs ASFMR1 and FMR4 are generated from the *FMR1* gene locus. ASFMR1 has multiple alternative splicing patterns and overlaps the 5′ untranslated region (UTR) CGG repeat region of *FMR1* [76]; FMR4 is initiated upstream of the *FMR1* start site. Alternative splicing of ASFMR1 seems to exhibit pre-mutation-specific profiles and is also silenced in FXS patients and upregulated in pre-mutation carriers, suggesting that a common process is responsible for regulating the expression of these transcripts. *FMR4* is also silenced in FXS patients because of a CGG expansion repeat in the 5′UTR of the *FMR1* gene and upregulated in pre-mutation carriers [77]; thus, their absence in the neurons of affected patients might contribute to the pathogenesis of this neurological disorder.

Another microsatellite expansion disease in which lncRNAs are involved is the spinocerebellar ataxia type 8 (SCA8), an autosomal dominant disorder, characterized by bidirectional transcription of this expansion repeat from opposite strands, forming both a protein-coding transcript encoding a polyglutamine expansion, ATXN8, and a lncRNA transcript containing a CUG expansion, ATXN8OS [78]. This suggests that SCA8 pathogenesis involves a toxic gain of function at both the protein and RNA levels. A recent study found that the expanded ATXN8OS transcript accumulates in ribonuclear inclusions in the GABAergic neurons of SCA8 patients [79]. These inclusions co-localized with splicing factor, MBNL1, altering the activity of MBNL alternative splicing proteins [79].

Huntington’s disease (HD) is caused by an expansion repeat mutation in the *Htt* gene, which encodes a ubiquitously expressed 3144 amino acid protein of unknown function, leading to a toxic gain of function in the mutant protein [80], which promotes aberrant nuclear-cytoplasmic trafficking of the master neuronal regulator REST. The result is the deregulation of REST target gene expression in tissues from animal models of HD and human HD, which include both protein-coding genes as well as ncRNAs, such as lncRNAs. It is therefore likely that HD tissues are also characterized by dysregulation of lncRNA expression. ChiP-seq data showed that the *HAR1* locus is under control of REST [81]. The *HAR1* region contains lncRNAs, *HAR1F* and *HAR1R*. HAR1F is specifically expressed in the neurons of the marginal zone during development of the cortex and in the frontal cortex, hippocampus, cerebellum, thalamus and hypothalamus in the adult brain [82]. The levels of HAR1F and HAR1R are decreased in HD brains compared with normal brains [83].

#### 6.1.2. LncRNAs in Neurodegenerative Diseases, such as Alzheimer’s Disease (AD)

β-site amyloid precursor protein-cleaving enzyme 1 (BACE1) is a crucial enzyme in AD pathophysiology, involved in the cleavage of the amyloid precursor protein (*APP*) and the generation of amyloid peptides which can aggregate and form plaques. Faghihi and colleagues characterized a conserved non-coding antisense transcript for BACE1, called BACE1-AS, which functions as a regulator of BACE1 gene expression. BACE1-AS upregulates BACE1 levels in response to a variety of stresses, including Aβ 1–42 exposure, and is elevated in several brain regions of patients with AD. These findings imply that BACE1-AS is deregulated in AD, which induces feed-forward regulation of BACE1, increases Aβ levels, and thus may promote the pathogenesis of the disease [84].

Mus *et al.* found a link between altered levels of a lncRNA, BC200, and AD [85]. Increased levels of BC200 were found in brain regions that are preferentially affected in AD, such as the hippocampus, which correlated with disease severity. Further, in advanced stages of AD, BC200 was mis-localized and clustered in the perikaryon. These observations suggest that deregulation of this synaptic lncRNA is involved in the synaptic and neural network dysfunction that is found in both early and later stages of AD [85].

Together with neurological diseases, a number of psychiatric disorders have also been associated with lncRNAs (Table 1). The risk of developing schizophrenia (SZ), schizoaffective disorder, bipolar disorder, major depression, and autistic spectrum disorders has been linked to the disruption of the *DISC* genomic locus, which encodes both the *DISC1* protein-coding gene and the *DISC2* lncRNA [86–88]. *DISC2* controls the expression of its partner, *DISC1*, which modulates multiple aspects of CNS structure and function such as embryonic and adult neurogenesis [89]. Another lncRNA, Gomafu, implicated in brain and retinal development [62,90] binds directly to the splicing factors QKI and SRSF1 and the dysregulation of Gomafu induces alternative splicing that resemble those observed in SZ for the archetypal SZ-associated genes *DISC1* and *ERB4*. Moreover, Gomafu is downregulated in post-mortem cortex of SZ subjects, suggesting a role in SZ pathogenesis for this lncRNA [91].

## 7. Conclusions and Future Perspectives

Several recent studies suggest that lncRNAs play a pivotal role in many key biological processes, although their mechanisms of action are yet to be fully elucidated. Currently there is great interest in identifying the functions of this novel class of transcripts. There is strong evidence that many lncRNAs are biologically relevant, with a large percentage of these molecules functioning through their interactions with chromatin-modifying complexes to alter gene expression [3]. These findings are beginning to shed light on how chromatin-modifying complexes are targeted to specific genomic loci and suggest the interesting idea that lncRNAs are in some way driving chromatin-modifying complexes to genomic loci.

LncRNAs have, in a relatively short period of time, become recognized as a major new class of genes that may potentially comprise a major component of the genome’s information content, complementary and comparable in abundance and complexity to the proteome. Furthermore lncRNAs have already been reported in a wide range of human diseases suggesting that their activity is crucial for human health.

In addition, therapeutic strategies that target endogenous mRNA molecules, such as those employing RNA interference (RNAi) and other customized oligonucleotide approaches with the capacity to reprogram disease-associated mRNAs, are now being developed [92]. These approaches could be adapted to target lncRNAs whose expression is dysregulated in CNS disorders. These observations suggest that lncRNAs represent a versatile class of factors that are centrally important to the modulation of different CNS processes and may represent a major layer underlying the genetic programming of brain development that could potentially be utilized for developing novel diagnostic and therapeutic tools for the cure of CNS disorders.

## Figures and Tables

**Table 1:** Examples of lncRNAs that are dysregulated in neurological disorders.

lncRNA	Disease association	Biological function	Reference
Ube3a-as	Angelman Syndrome	Repress paternal *Ube3a* expression	[72]
ASFMR1 FMR4	Fragile X Syndrome	Regulate the expression of *ASFMR1* and *FMR1* genes	[76,77]
ATXN8OS	SCA8	Alteration of the activity of the splicing factor *MBNL1*	[78,79]
HAR1F HAR1R	Hungtington’s Disease	Influence genes promoting aberrant nuclear-cytoplasmatic trafficking of *REST* gene	[83]
BC200 BACE1-AS	Alzheimer’s	Involved in the synaptic and neural network dysfunction Regulates *BACE1* gene expression	[84,85]
DISC2 Gomafu	Psychiatric disorders	Controls the expression of *DISC1*	[86–89]
		Implicated in brain and retinal development	[91]
